# The Fruits of *Paris polyphylla* Inhibit Colorectal Cancer Cell Migration Induced by *Fusobacterium nucleatum*-Derived Extracellular Vesicles

**DOI:** 10.3390/molecules26134081

**Published:** 2021-07-04

**Authors:** Liang-Tzung Lin, Yeu-Ching Shi, Chen-Yen Choong, Chen-Jei Tai

**Affiliations:** 1 Department of Microbiology and Immunology, School of Medicine, College of Medicine, Taipei Medical University, Taipei 110301, Taiwan; ltlin@tmu.edu.tw; 2 Graduate Institute of Medical Sciences, College of Medicine, Taipei Medical University, Taipei 110301, Taiwan; jasmineycs@yahoo.com.tw; 3 Department of Chinese Medicine, Taipei University Hospital, Taipei 110301, Taiwan; chenyen@tmu.edu.tw; 4 Traditional Herbal Medicine Research Center, Taipei Medical University Hospital, Taipei 110301, Taiwan; 5 Department of Obstetrics and Gynecology, School of Medicine, College of Medicine, Taipei Medical University, Taipei 110301, Taiwan

**Keywords:** extracellular vesicles, *Fusobacterium nucleatum*, complementary medicine, cell migration, *Paris polyphylla*

## Abstract

Colorectal cancer (CRC) is one of the most common cancers worldwide. Gut microbiota are highly associated with CRC, and *Fusobacterium nucleatum* was found to be enriched in CRC lesions and correlated with CRC carcinogenesis and metastases. *Paris polyphylla* is a well-known herbal medicine that showed anticancer activity. The present study demonstrates that *P. polyphylla* inhibited the growth of CRC cells. In addition, treating with active compounds pennogenin 3-*O*-beta-chacotrioside and polyphyllin VI isolated from *P. polyphylla* inhibited the growth of *F. nucleatum*. We also found that extracellular vesicles (EVs) released from *F. nucleatum* could promote mitochondrial fusion and cell invasion in CRC cells, whereas active components from *P. polyphylla* could dampen such an impact. The data suggest that *P. polyphylla* and its active ingredients could be further explored as potential candidates for developing complementary chemotherapy for the treatment of CRC.

## 1. Introduction

*Paris polyphylla* is a well-known Asian herbal medicine that is commonly used in Taiwan and China to treat headache, fever, burn, wound, and snake poisoning [1]. The extract of *P. polyphylla* and its active components have been shown to have anticancer activity both in vitro and in vivo. Numerous natural steroidal saponins isolated from herbs have shown the potential on apoptosis-promoting activity against several cancer cell types [2,3,4,5]. *P.* *polyphylla* treatment can also inhibit epithelial–mesenchymal transition (EMT) and invasion of breast cancer [6] and lung cancer cells [3,4,5]. Our previous work also demonstrates that extract of *P. polyphylla* inhibits the growth of ovarian carcinoma cells through inducing apoptosis and suppresses EMT and mitochondrial activity via the downregulating peroxisome proliferator-activated receptor-gamma coactivator (PGC)-1alpha [7].

Gut microbiota have been reported to interact with CRC, and different bacterial communities may exist across stages of colorectal carcinogenesis [8,9,10,11]. Among the frequently identified bacteria, the association of *F. nucleatum* and CRC is widely acknowledged [11,12]. The presence of *F. nucleatum* has been found to promote CRC tumorigenesis, drug resistance, metastasis, and modulate tumor microenvironment [13,14,15,16,17]. In addition, gavage of fecal samples from patients with CRC promotes intestinal carcinogenesis in germ-free and conventional mice [18]. Another study also showed that inhibition of *F. nucleatum* by natural compound could improve negative microbiota and rescue the tumor microenvironment in CRC [19].

Extracellular vesicles (EVs) derived from bacteria are important in host and microbe communication [20]. Bacterial EVs have become more relevant as a possible important agent for mediating host–pathogen interactions. In addition to the negative impact of pathogenic bacteria for tumorigenesis, such gut microbe-derived EVs have also been found to result in disease and induction of tumorigenesis in intestinal epithelium [21,22]. In this study, we investigated the potential effects of the ethanolic extracts from the fruits of *P. polyphylla* (EEFPP) on the suppression of colorectal carcinoma cells with *F. nucleatum*-derived EVs.

## 2. Results and Discussion

### 2.1. Treatment Effect of P. polyphylla on Colorectal Cancer Cell Growth

**Figure 1:**
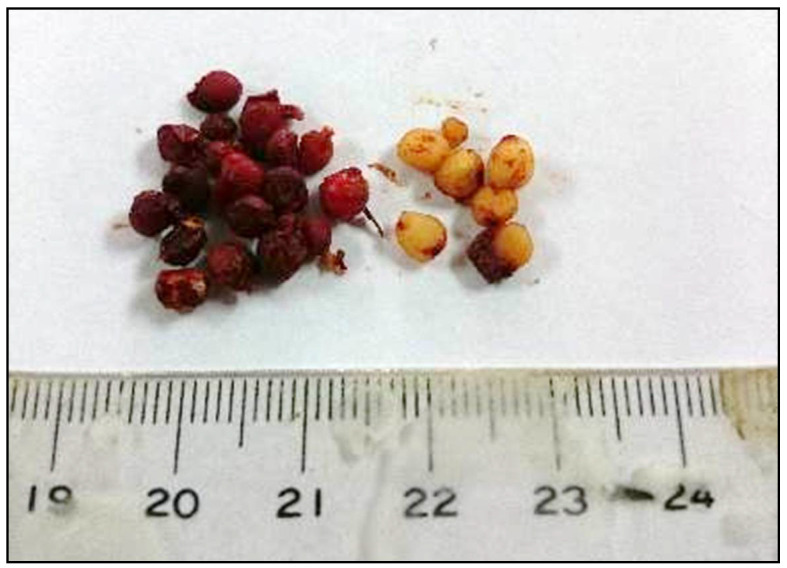
The morphology of fruits obtained from *P. polyphylla*.

**Figure 2:**
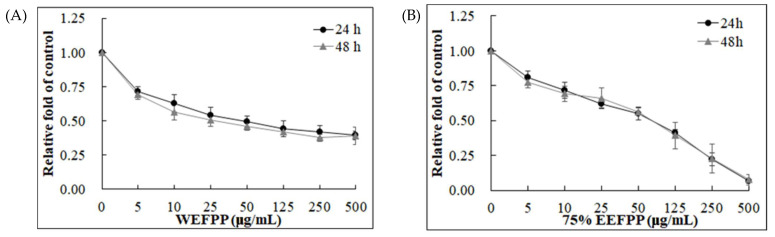
Cell viability of Caco-2 colorectal carcinoma cells after treatment with WEFPP (**A**) or EEFPP (**B**) for 24 h and 48 h. Data are shown as means ± SD (*n* = 3).

**Figure 3:**
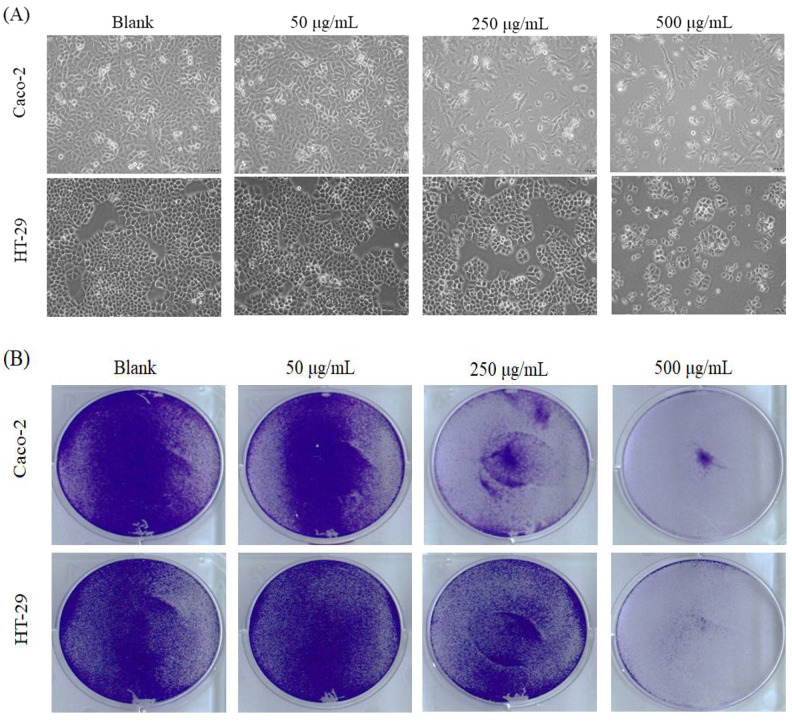

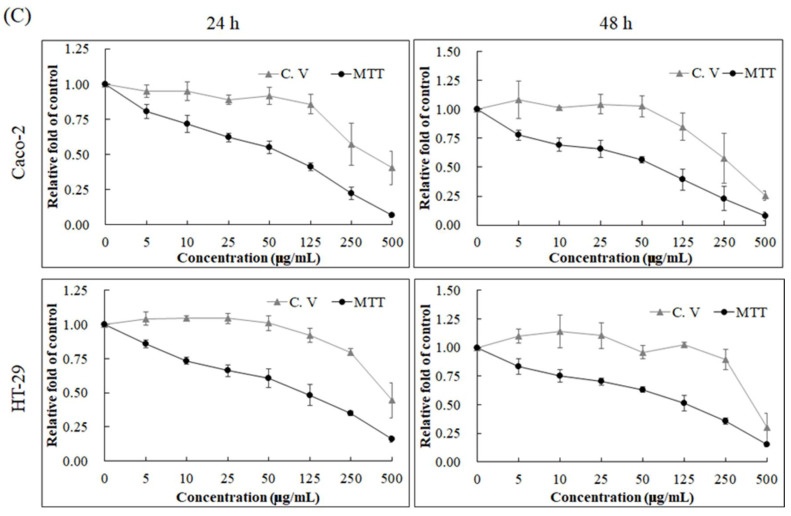
Effect of EEFPP on the growth in Caco-2 and HT-29 colorectal cancer cells. (**A**) Effects of EEFPP on cell morphology after 24 h treatment. (**B**) Crystal violet staining of cells after EEFPP treatment for 24 h. (**C**) Quantitative analysis of MTT and crystal violet staining (C. V) in cells treated with EEFPP for 24 h and 48 h. Data are shown as means ± SD (*n* = 3). MTT data of Caco-2 are adopted from Figure 2B.

The fruits of *P. polyphylla* are shown in Figure 1, and the extraction yields were 21.09% and 20.14% in water extracts and 75% in ethanol extracts, respectively (data not shown). The suppression of Caco-2 colorectal cancer cells treated by water extracts (WEFPP) or 75% ethanolic extracts (EEFPP) from fruits of *P. polyphylla* for 24 h or 48 h was investigated by 3-(4,5-dimethylthiazol-2-yl)-2,5-diphenyltetrazolium bromide (MTT) assay. Results indicated that EEFPP exerted effective potential for lowering viability of Caco-2 cells than WEFPP (Figure 2). Morphologically, we found that EEFPP treatment for 24 h resulted in cell shrinkage at 250 and 500 μg/mL in Caco-2 cells. By contrast, another colorectal carcinoma cell line, HT-29, was less affected in its morphology by EEFPP treatment (Figure 3A). Since cell viability measured by MTT assay is dependent on cellular metabolic activity, we further validated the suppression of Caco-2 and HT-29 cells treated by EEFPP with crystal violet staining (Figure 3B). Results indicated that EEFPP caused apparent cytotoxic effects at 250 and 500 μg/mL in Caco-2 cells and HT-29 cells, respectively, after 24 h and 48 h as demonstrated by both MTT assay and CV staining (Figure 3C).

### 2.2. Effect of P. polyphylla on the Growth of Fusobacterium nucleatum and Clostridium difficile

**Figure 4:**
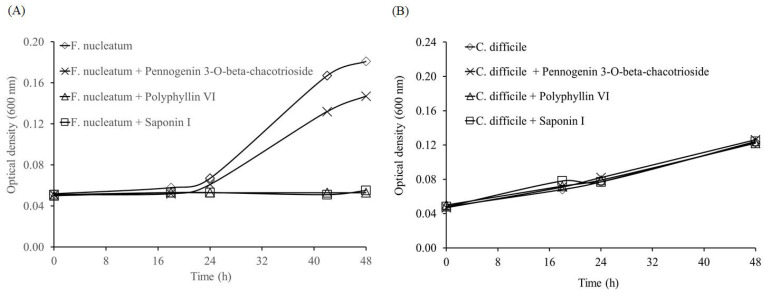
Effect of pennogenin 3-*O*-beta-chacotrioside, polyphyllin VI, and saponin I at 5 μM on the growth curve of (**A**) *Fusobacterium nucleatum* and (**B**) *Clostridium difficile*.

*F. nucleatum* was reported to promote invasion, migration, and drug tolerance of CRC cells [13,14,15,16,17]. In addition, a higher prevalence of *C. difficile* was also found in CRC lesions in comparison to normal tissues [23]. Therefore, we then evaluated the effect of active compounds in *P. polyphylla* on these two pathogens. Previously, we identified two saponins, pennogenin 3-*O*-beta-chacotrioside and polyphyllin VI [24], from the ethanol extract of *P. polyphylla* [25]. Here, we also included saponin I, another commonly isolated saponin from the rhizomes of *P. polyphylla* [26], for comparison. All compound treatments inhibited *F. nucleatum* growth, with polyphyllin VI and the saponin I being the most effective, but none of these compounds were effective in suppressing *C. difficile* (Figure 4).

**Figure 5:**
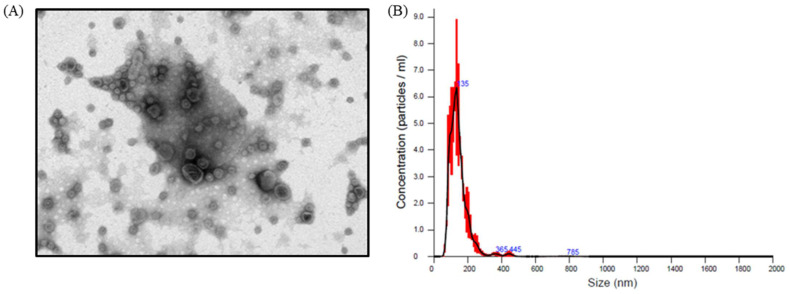
The characteristics of extracellular vesicles (EVs) derived from *F. nucleatum*. (**A**) Morphology of EVs by transmission electron microscope and (**B**) size distribution of EVs by nanoparticle tracking analysis.

**Figure 6:**
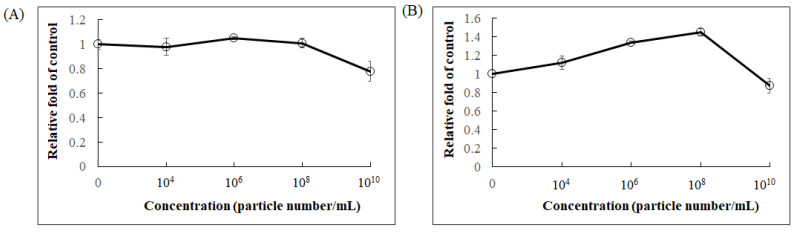
The effect of *F. nucleatum*-derived EVs on cell proliferation and cell viability. (**A**) Cell survival by crystal violet stain and (**B**) mitochondrial activity by MTT measurement in Caco-2 cells after 36 h treatment. Data are shown as means ± SD (*n* = 3).

Extracellular vesicles (EVs) are membrane-based structures which can carry various types of cellular components such as lipids, proteins, and nucleic acids. The effects of EVs can vary. *Escherichia coli*-derived EVs were shown to promote immunity in a mouse model to attenuate cancer progression [27], whereas *Staphylococcus aureus*-derived EVs caused cell damage and death [28]. It has been reported that *F. nucleatum*-derived EVs induce inflammation via TLR2 pathway in intestinal epithelial cells (IECs), and these effects are similar to those observed with whole *F. nucleatum* bacteria on IECs [29]. Other studies also demonstrated that *F. nucleatum*-derived EVs markedly promoted epithelial barrier loss and oxidative stress in Caco-2 cells [30] and induced intestinal inflammation [31]. We first isolated EVs from *F. nucleatum* and investigated their effect on CRC proliferation. The morphology of *F. nucleatum*-derived EVs was observed by electron microscopy, and their particle size ranged from 100–200 nm (Figure 5). When Caco-2 cells were treated with the purified EVs, cell viability was not affected at concentrations up to 10^8^ particles/mL, as determined by crystal violet staining (Figure 6A). However, cell metabolic activity measured by MTT assay increased dose-dependently between concentrations from 10^4^ to 10^8^ particles/mL (Figure 6B), suggesting that *F. nucleatum*-derived EVs may modulate mitochondrial dynamics.

### 2.3. Active Compounds Isolated from Fruits of P. polyphylla Inhibited F. nucleatum EV-Induced Mitochondrial Fusion and Migration in Caco-2 Cells

**Figure 7:**
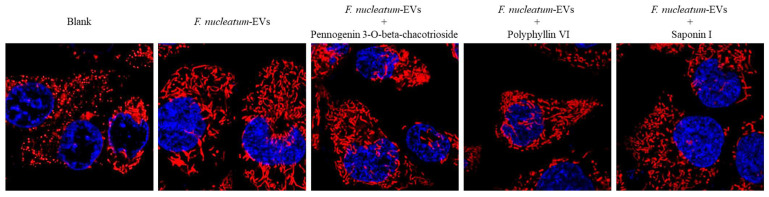
Mitochondria morphology of Caco-2 cells after treating with *F. nucleatum*-derived EVs (1 × 10^8^ particles/mL) for 36 h with or without pennogenin 3-*O*-beta-chacotrioside, polyphyllin VI, and saponin I at 1 μM.

Next, we investigated the role of *F. nucleatum*-derived EVs on mitochondrial function in Caco-2 cells. Mitochondrial dynamics are regulated by mitochondrial fusion and fission. Mitochondrial fission often occurs during apoptosis [32] and autophagy [33], whereas mitochondrial fusion promotes cell survival [34]. When Caco-2 cells were treated with *F. nucleatum*-derived EVs, we found an increased mitochondrial fusion (Figure 7, EVs), which may have contributed to the enhanced MTT readout. We then evaluated the effect of pennogenin 3-*O*-beta-chacotrioside and polyphyllin VI in Caco-2 cells treated with *F. nucleatum*-derived EVs and found that pennogenin 3-*O*-beta-chacotrioside and polyphyllin VI appeared to attenuate mitochondrial fusion, similar to the saponin I (Figure 7).

**Figure 8:**
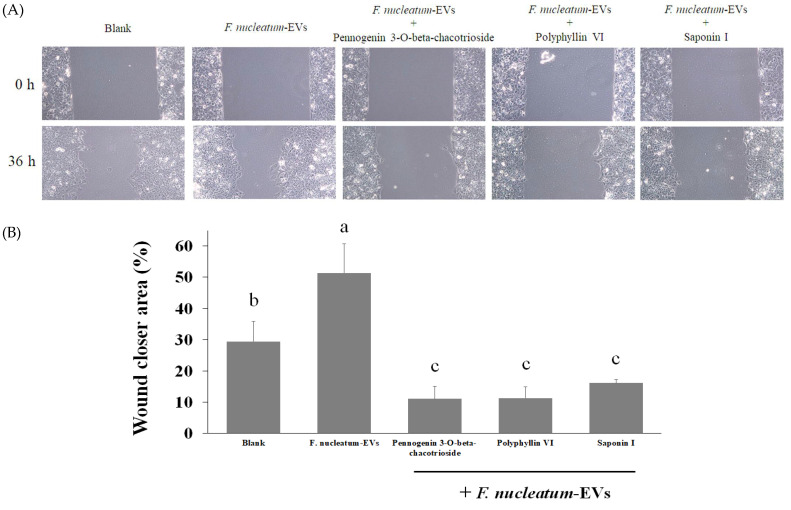
Cell migration capacity was determined by wound healing assay. Caco-2 cells were treated with *F. nucleatum*-derived EVs (1 × 10^8^ particles/mL) for 36 h with or without pennogenin 3-*O*-beta-chacotrioside, polyphyllin VI, and saponin I at 1 μM. Cells were photographed after incubating for 36 h (**A**) and the area of the wound was quantified (**B**). Data are shown as means ± SD (*n* = 3). Variables that are statistically different (*p* < 0.05) are denoted by different letters.

In addition, given that *Fusobacterium spp.* were found in the metastatic lesions of CRC [35] and potentially stimulate CRC migration and invasion [36], we further examined whether *F. nucleatum*-derived EVs have a similar effect. As shown in Figure 8 with a wound-healing assay of Caco-2 cells, *F. nucleatum*-derived EVs enhanced cell migration for an average of 20%. Treatment of these EV-incubated cells with active compounds from the fruits of *P. polyphylla* (pennogenin 3-*O*-beta-chacotrioside, polyphyllin VI) and saponin I successfully inhibited wound closure area to below basal level, indicating the antimigration effect of these compounds. Taken together, active compounds isolated from *P. polyphylla* may have the potential to regulate the negative impacts of *F. nucleatum*-derived EVs on CRC.

### 2.4. Effect of P. polyphylla on the Growth of Akkermansia muciniphila and Bifidobacterium bifidum

**Figure 9:**
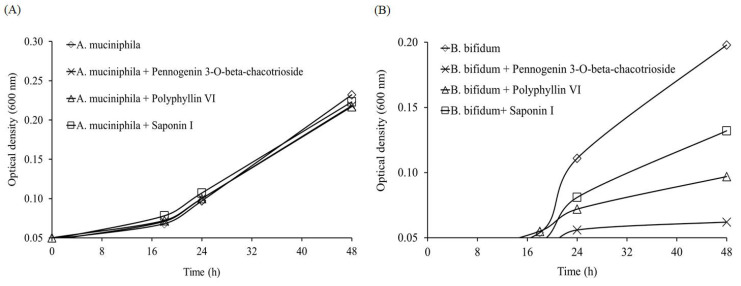
Effect of pennogenin 3-*O*-beta-chacotrioside, polyphyllin VI, and saponin I (5 μM) on the growth curve of (**A**) *Akkermansia muciniphila* and (**B**) *Bifidobacterium bifidum*.

Finally, to assess the feasibility of using *P. polyphylla* components for the treatment of CRC, we examined whether the abovementioned compounds affect common probiotics in human gut microbiota, such as *Akkermansia* *muciniphila* and *Bifidobacterium bifidum*. *A. muciniphila* mainly relies on the mucus secreted by human intestinal cells as essential nutrients. Studies found that a decrease in *A. muciniphila* may result in dysbiosis in intestinal microbial ecology [37], and *A. muciniphila* has been proven to improve obesity and diabetes [38,39] and increase the success rate of programmed death-1 (PD-1)-based immunotherapy in cancer patients [40]. On the other hand, a recent study indicated that *B. bifidum* could increase the expression of tumor suppressor genes and inhibit oncogenes to improve CRC treatment [41]. These studies highlight the importance of probiotics in CRC treatment. As shown in Figure 9, the growth of *A. muciniphila* was not affected by any of the tested compounds, but the growth of *B. bifidum* was inhibited by pennogenin 3-*O*-beta-chacotrioside, polyphyllin VI, and saponin I to different extents. These results revealed that supplement of *B. bifidum* may be necessary to compensate its inhibition caused by *P. polyphylla* when the herb’s components are used as a complementary therapy of CRC.

## 3. Materials and Methods

### 3.1. Chemicals

Fruits of *P. polyphylla* were purchased from Taiwan Indigena Botanica Co., Ltd. (Taipei, Taiwan), and 10 g of the material was extracted with ethanol (100 mL) three times at room temperature for 24 h. After evaporating the solvents through freeze-drying, a residue was obtained and stored at −20 °C. Crystal violet and trypsin were purchased from Sigma Chemical Co. (St. Louis, MO, USA). Fetal bovine serum (FBS) was purchased from Life Technologies (Auckland, New Zealand). Dimethyl sulfoxide was purchased from Wako Pure Chemical Industries (Saitama, Japan). Pennogenin 3-*O*-beta-chacotrioside was purchased from BioCrickBioTech (Chengdu, Sichuan, China). Polyphyllin VI was purchased from Chem Faces (Wuhan, Hubei, China). Saponin I was purchased from Xibao Biotechnology Co., Ltd. (Shanghai, China).

### 3.2. Method of Isolation and Identification of Active Compounds

Firstly, 50 g of *Paris polyphylla* was dissolved in 1 L of 100% ethanol and extracted. The extracts were then separated using an ODS (octadecylsilyl) column into different parts. After eluting with different concentrations of methanol, 80% methanol-treated parts were isolated and detected by HPLC.

### 3.3. Cell Culture

Human CRC cell lines Caco-2 and HT-29 (Bioresource Collection and Research Center, HsinChu, Taiwan) were grown in Dulbecco’s modified Eagle’s medium (Gibco BRL, Grand Island, NY, USA) containing 2 mM L-glutamine and 1.5 g/L sodium bicarbonate, supplemented with 10% FBS and 2% penicillin–streptomycin (10,000 U/mL penicillin and 10 mg/mL streptomycin). The cells were cultured in a humidified incubator at 37 °C with 5% CO_2_.

### 3.4. Cell Viability Assessment

The cytotoxic effects of WEFPP and EEFPP in Caco-2 and HT-29 cells were measured using MTT assay and crystal violet staining assay. Cells were seeded on 24-well plates (3 × 10^4^ cells per well) and treated with various concentrations of test extracts for 24 h or 48 h. For MTT assay, MTT solution was added to each well at the endpoint for an additional 4 h incubation at 37 °C. After the addition of DMSO, absorbance value of formazan was measure at 570 nm. For crystal violet staining, the medium was removed at the endpoint, and cells were washed with phosphate-buffered saline (PBS), stained with 2 g/L crystal violet in phosphate-buffered formaldehyde for 20 min, and then washed with water. Crystal violet-stained cells were dissolved in 20 g/L SDS solution, and its absorbance was measured at 600 nm.

### 3.5. Mitochondrial Fusion and Fission

Mitochondrial staining was performed as previously described [42]. In brief, cells were cultured with serum-free culture medium containing 250 nM Mitotracker Deep-Red FM (Invitrogen, Waltham, MS, USA) for 30 min and washed twice by PBS, and then the cell nuclei were incubated with Hochest 33342 stain for 10 min. Mitochondrial morphology was then observed using a confocal microscope.

### 3.6. Bacterial Culture

*Fusobacterium nucleatum* (BCRC17680), *Clostridium difficile* (BCRC80997), *Akkermansia muciniphila* (BCRC81048), and *Bifidobacterium bifidum* (BCRC14630) were all purchased from Bioresource Collection and Research Center (HsinChu, Taiwan). *F. nucleatum* and *C. difficile* were grown in tryptic soy agar with 5% defibrinated sheep blood at 37 °C. *A. muciniphila* was grown in Chocolate II (BBL) broth at strictly anaerobic condition at 37 °C, and *B. bifidum* was grown in MRS broth at 37 °C.

### 3.7. Isolation and Characterization of F. nucleatum-Derived EVs

F. nucleatum-derived EVs were isolated using a previously reported ultracentrifugation protocol [43]. In brief, F. nucleatum culture at stationary phase was centrifuged at 3000× *g* for 15 min to remove the cells, and the supernatant was subsequently centrifuged at 35,000× *g* for 60 min to remove bacterial debris and organelles. The supernatant was then subjected to ultracentrifugation at 200,000× *g* for 60 min, and the obtained EVs pellets were resuspended in PBS and passed through 0.22 μm filter to obtain pure EVs. The morphology, number, and size distribution of EVs were analyzed by transmission electron microscope (TEM) and Nano-ZS 90 dynamic light scattering nanoparticle tracking analysis (Malvern, UK).

### 3.8. Wound-Healing Assay

A wound-healing assay was used to determine cell migration ability. After growing to 80% confluence in 24-well plates, wounds were made in Caco-2 cells via scraping the monolayer using a plastic pipette tip. The cells were then washed three times by PBS to remove debris and treated with *F. nucleatum*-derived EVs (1 × 10^8^ particles/mL) with or without compounds (pennogenin 3-*O*-beta-chacotrioside, polyphyllin VI, and saponin I) and incubated at 37 °C with serum-free medium. Cells were photographed after incubating for 36 h. All experiments were performed in triplicate. The area of the wound was measured with Image J software (NIH, Bethesda, MD, USA).

### 3.9. Statistical Analysis

Results were expressed as means ± SD. Comparisons among groups were made using one-way ANOVA. The differences between mean values in all groups were tested through Duncan’s multiple-range test (SPSS statistical software package, version 17.0, SPSS, Chicago, IL, USA). A *p*-value less than 0.05 was considered significant.

## 4. Conclusions

The present study demonstrated that *P. polyphylla* and its active components inhibited the growth of CRC cells. In addition, growth of gut microbe *F. nucleatum*, which was demonstrated to positively correlate with the development of CRC, could be inhibited by treatment with the active compounds pennogenin 3-*O*-beta-chacotrioside and polyphyllin VI isolated from *P. polyphylla*. Our data showed that EVs released from *F. nucleatum* induced mitochondrial fusion and promoted cell migration in CRC cells, whereas the two active components isolated from *P. polyphylla* could reverse these effects. We suggest that *P. polyphylla* and its active ingredients could be further explored as potential candidates for the development of complementary chemotherapy against CRC.

## Data Availability

Data is contained within the article.

## Sample Availability

All compounds are commercially available as indicated in the text.

## Abbreviations

CRC	colorectal cancer
EEFPP	ethanolic extracts isolated from fruits of *Paris polyphylla*
EV	extracellular vesicle
WEFPP	water extracts isolated from fruits of *Paris polyphylla*
